# Glaucoma in Early Life

**Published:** 1890-05-03

**Authors:** 


### GLAUCOMA IN EARLY LIFE.

Dr. Laqueur, of Strassburg, has met with six cases of glaucoma in persons far below the age at which this disease is usually observed. Four of them were males, aged 17, 23, 25, and 36 years, and two were women of 17 and 24 years respectively. Such cases are not unknown, but the vast majority of sufferers are over 45 or 50 years. In one only had there been any irritative prodromata, the disease being simple in the remaining five. In all it was unilateral, but in one the other eye was affected later. They complained of cloudy vision, with coloured rings.

From these cases he concludes that glaucoma occurring in young adults differs from that seen in more advanced life in the anterior chamber not being flattened, indeed rather the reverse, the iris being normal and the pupil sensitive to light or fairly so, and the globe not so hard and tense, though the "cup" was well marked in all.

